# Parenting Stress, Parenting Self‐Efficacy and Dyadic Coping in Parents of Preterm Infants in China: A Dyadic Analysis

**DOI:** 10.1002/nop2.70700

**Published:** 2026-07-17

**Authors:** Yuying Mo, Wanxiang He, Xiaojuan Liu, Wei Xia

**Affiliations:** ^1^ Peking University Shenzhen Hospital Shenzhen Guangdong China; ^2^ School of Nursing Sun Yat‐Sen University Guangzhou Guangdong China

**Keywords:** actor‐partner interdependence mediation model, dyadic coping, parenting self‐efficacy, parenting stress, parents of preterm infants

## Abstract

**Aim:**

To examine actor and partner effects of parenting stress on self‐efficacy mediated by dyadic coping among Chinese parents of preterm infants.

**Design:**

A cross‐sectional study was conducted from November 2022 to March 2024, involving 120 parental dyads of preterm infants discharged from Neonatal Intensive Care Units (NICU) in two tertiary hospitals in China. Parenting stress, self‐efficacy and dyadic coping were assessed. Multiple regression and Actor‐Partner Interdependence Mediation Model (APIMeM) were performed.

**Results:**

Mothers and fathers reported similar levels of parenting stress and self‐efficacy, but mothers scored significantly higher in dyadic coping. Parenting self‐efficacy was associated with stress from observations of the infant's appearance/behaviour, NICU environmental stress, common coping and perceived partner support. Positive coping mediated the relationship between parenting stress and self‐efficacy for mothers but not for fathers.

**Conclusions:**

Positive coping buffered the stress‐self‐efficacy link only for mothers, revealing distinct gendered coping patterns. Parenting self‐efficacy was directly associated with infant appearance and NICU‐related stress, common coping and partner support. Couple‐based NICU interventions should be tailored to gender‐specific coping mechanisms. Nurses can promote family‐centred, gender‐sensitive support, including reducing environmental stressors, addressing appearance‐related stress, encouraging common coping and fostering partner support to enhance parental self‐efficacy.

**Reporting Method:**

Strengthening the Reporting of Observational Studies in Epidemiology guidelines.

**Public Contribution:**

No patient or public contribution.

**Implications for the Profession and Patient Care:**

Based on our findings, interventions should focus on reducing stress related to the infant's appearance and the NICU environment, promoting common coping strategies, and strengthening mutual partner support. For mothers, enhancing positive coping may be particularly beneficial, as it mediated the stress‐self‐efficacy relationship. For fathers, efforts to reduce avoidant coping and encourage active involvement in infant care are recommended. By addressing these specific factors, NICU teams can enhance parenting self‐efficacy and foster balanced family adaptation, ultimately improving outcomes for preterm infants and their families.

**Trial Registration:**

The protocol of the study was registered at ClinicalTrials.gov (NCT05592210) on March 27, 2023

## 1 Background

According to reports from the World Health Organization, the global preterm birth rate stands at 10.6%, with approximately 14.84 million preterm births annually (Deng et al. 2021). Nearly 1.2 million preterm infants are born annually in China (Chawanpaiboon et al. 2019). Birth defects are three times more common in premature infants than full‐term (Lutkiewicz 2020). Furthermore, preterm infants are at an elevated risk of both immediate and long‐term complications. Immediate complications include intracranial haemorrhage, atelectasis, pneumonia and gastrointestinal bleeding. Long‐term complications encompass bronchopulmonary dysplasia and severe brain injury (Mowitz et al. 2022). Parental behaviour exerts a significant influence on the growth and development of premature infants, and proactive parental engagement can facilitate their healthy development (Madigan et al. 2019). Nevertheless, preterm infants' inherent physiological immaturities, such as underdeveloped sucking patterns, pose persistent challenges post‐discharge, including feeding difficulties, weight loss and the need for home oxygen therapy (Landsem and Handegård 2024). These factors frequently lead to parents experiencing a sense of being overwhelmed and a diminished sense of self‐efficacy during the transition period (Khajehei and Lee 2019). Raising children is a shared responsibility; parents need to collaborate and support each other to minimize negative parenting behaviours (Wang, Yao, et al. 2025). Therefore, parenting behaviours in preterm infants require greater attention.

Parenting stress refers to the pressure parents experience while raising their children and is influenced by factors such as personality traits, parent–child interactions, child characteristics and family situations (Fang et al. 2024). Preterm birth undoubtedly places significant stress on the parents and families of preterm infants; trauma and stress persist even after discharge from the Neonatal Intensive Care Unit (NICU) (Griffith et al. 2022). Research has revealed that nearly one‐third of mothers of preterm babies continue to experience grief for up to 9 months after delivery (Calderon‐Noy and Gilboa 2021). Parenting stress can impact parents' overall health and parenting style (Landsem and Handegård 2024). Excessive parenting stress can negatively impact parental abilities and diminish self‐efficacy. Additionally, positive parenting behaviour correlates with high parenting self‐efficacy, which can facilitate mothers' role transformation (Sæther et al. 2022), enabling them to exhibit better parenting behaviour and parent–child interactions (Seetharaman et al. 2022). Mothers with low parenting self‐efficacy often struggle to apply effective parenting knowledge in practice (Botha et al. 2020). Therefore, they often adopt negative coping styles when confronted with parenting challenges. Meanwhile, within the family stress model framework, parenting self‐efficacy has been identified as a resilience factor that buffers the negative cascade from external stressors to parenting outcomes (Chen et al. 2025). Most current studies on parenting self‐efficacy of preterm parents assess the effects of care interventions (Koliouli et al. 2022), while few focus on preterm fathers. Therefore, research on both parents of preterm infants is essential for improving their parenting self‐efficacy and behaviours.

Previous studies posited that stress within a couple is interdependent and invariably affects both partners in the relationship. The most effective stress management occurs within a dyadic framework (Landolt et al. 2023). Moreover, research indicates that the higher a couple's sense of self‐efficacy, the stronger their dyadic coping abilities (Liu et al. 2024). Dyadic coping refers to shared reactions and strategies used by both partners in response to stressful events (Landolt et al. 2023). However, few studies have explored the relationship between dyadic coping and parenting self‐efficacy. Wang, Xu, et al. (2025) research on the parents of children with leukaemia indicated that parents with a lower dyadic coping score tend to reduce their parenting self‐efficacy. In a study on dyadic relationships, Oltra‐Benavent et al. (2019) identified a significant positive correlation between parenting self‐efficacy and dyadic adjustment. Hadian Shirazi et al. (2022) demonstrated that when fathers are trained to support their wives, it relieves maternal stress, improves mothers' parenting self‐efficacy, and has a direct positive effect on the care provided to their premature newborns. However, research on dyadic coping among parents of preterm infants and its interaction with parenting self‐efficacy remains limited in China, and a study examining the impact of dyadic coping on parenting stress and self‐efficacy in this population is rare.

Raising preterm infants is a shared responsibility requiring mutual cooperation and support. Within the Chinese sociocultural context, caregiving practices for preterm infants are situated within an intergenerational, collaborative family support system. This system features maternal‐centred daily care, sustained involvement of grandparents and progressively increasing father engagement (Sun et al. 2024). Research on parents of preterm infants has primarily focused on anxiety and depression, with limited attention to couples' parenting stress. Although studies have shown that parents with stronger self‐efficacy exhibit greater dyadic coping abilities (Liu et al. 2024), the relationship between dyadic coping and parenting self‐efficacy in this population remains unclear. Therefore, this study aimed to investigate how couples' parenting stress affects their own and their partner's parenting self‐efficacy through dyadic coping. We hypothesized that: (1) parenting stress would be negatively correlated with both actor and partner parenting self‐efficacy, (2) dyadic coping would be positively correlated with both actor and partner parenting self‐efficacy, and (3) dyadic coping would mediate the association between parenting stress and parenting self‐efficacy.

### 1.1 Conceptual Framework

**FIGURE 1:**
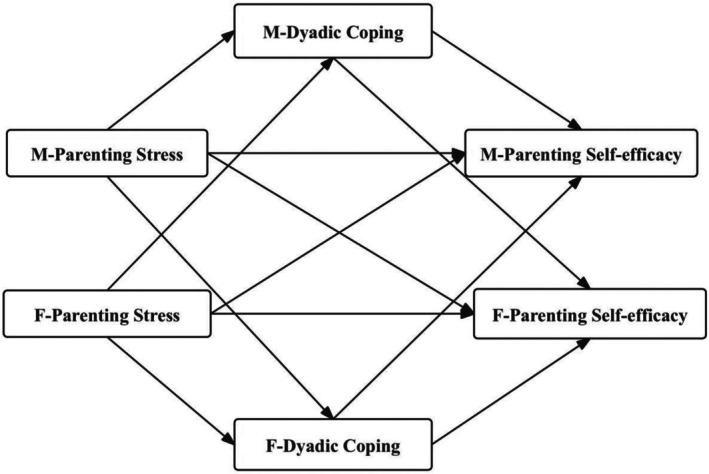
Conceptual framework of the study.

This study was guided by the Actor‐Partner Interdependence Model extended to Mediation (APIMeM) (Ledermann et al. 2011) and the study hypothesis to explore the mechanism of dyadic coping between parenting self‐efficacy and parenting stress in parents of preterm infants. As shown in the conceptual model (Figure 1), parenting self‐efficacy in parents of preterm infants is represented by the dependent variables *Y* and *Y*′, while parenting stress for both parents is denoted by *X* and *X*'. These measures are assessed separately for fathers and mothers, allowing for the examination of their correlation to represent partnership dynamics, which, in turn, can be used to predict or explain *Y* and *Y*′. According to the hypotheses of this study, parents' dyadic coping serves as a mediating variable between parenting stress and self‐efficacy (Figure 1).

## 2 Methods

### 2.1 Study Design and Sampling

A cross‐sectional study was conducted from November 2022–March 2024 using convenience sampling with parents whose newborns met the diagnostic criteria for preterm infants and were hospitalized in NICUs at two tertiary hospitals in Guangdong and Guangxi provinces, China. The preterm infants and their parents who met the inclusion criteria were invited to participate in this study.

The infants' inclusion criteria were as follows: (1) gestational age < 37 weeks, (2) admission to the neonatology department or NICU and (3) successful discharge after treatment. We excluded infants with incurable congenital malformations that impaired their quality of life and those who discontinued treatment due to medical conditions, familial circumstances, hospital transfers or fatalities.

The parents' inclusion criteria were as follows: (1) parents of the aforementioned eligible preterm infants with a gestational age < 37 weeks, (2) both aged ≥ 18 years, and (3) proficient in Chinese to complete questionnaires independently or with assistance from investigators. We excluded couples in which either partner had a documented clinical diagnosis of any psychiatric disorders recorded in the hospital medical record, including but not limited to major depressive disorder or anxiety disorders, or was receiving psychiatric treatment requiring hospitalization.

According to previous literature, the average preterm infant mother's parenting self‐efficacy score was 40.0, with a standard deviation of 3.0, an allowable error of 0.2 S (0.60), the average preterm infant father's parenting self‐efficacy score was 25.5, with a standard deviation of 3.7, an allowable error of 0.2 S (0.74) (Landsem and Handegård 2024), and a significance level (*α*) of 0.05. The required sample size was 97, according to the formula below:

$$N=z_{a}^{2}\frac{\sigma^{2}}{\delta^{2}}=1.96^{2}\times \frac{3^{2}}{0.6^{2}}=96.04\;\left(\text{Mother}\right)$$

$$N=z_{a}^{2}\frac{\sigma^{2}}{\delta^{2}}=1.96^{2}\times \frac{3.7^{2}}{0.74^{2}}=96.04\;\left(\text{Father}\right)$$

Considering a 20% invalid questionnaire rate, a minimum sample size of 120 pairs of parents of preterm infants was needed for this study.

### 2.2 Measures

A standardized questionnaire was used to collect parental demographics (including age, education level, occupation and monthly income), couple characteristics (including family annual income, fertility history, and number of children), and infant characteristics (gestational age, gender, and birth weight). Parental stress, parenting self‐efficacy and dyadic coping were assessed using validated scales for reliability and validity. The scale information is as follows.

#### 2.2.1 Parental Stressor Scale: NICU (PSS: NICU)

Parenting stress was measured using the PSS: NICU, compiled by Margaret Miles et al. (1993) in 1993. It contains 26 items divided into three dimensions, including the appearance and behaviour of infants (13 items), change in parental role (seven items), and visual and auditory perception of parents in the NICU (six items). Likert 5‐level scoring methods were used, with total scores ranging from 26 to 130. A higher score indicates a higher exposure level of parents to stressors. The Cronbach's *α* of the PSS: NICU in Chinese was 0.856 (Li et al. 2020).

#### 2.2.2 Karitane Parenting Confidence Scale (KPCS)

Parenting self‐efficacy was measured using the KPCS developed by Crncec et al. (2008) in 2008. The scale includes three dimensions: parenting, support and sense of competence, comprising 15 items. A four‐point Likert scale was adopted, with a total of 45 points. Higher scores indicated higher levels of parenting confidence. The Cronbach's *α* of the KPCS in Chinese was 0.769 (Pan et al. 2021).

#### 2.2.3 Dyadic Coping Inventory (DCI)

Parental dyadic coping was measured using the DCI, developed by Bodenmann (2008). The questionnaire contained 35 items in two major dimensions: positive and negative coping. A five‐point Likert scale was adopted, where higher total scores indicated more supportive partner interactions. Positive coping comprised 27 items (including Stress Communication, Emotion‐Focused Supportive dyadic coping, Problem‐Focused Supportive dyadic coping, Delegated dyadic coping and Common dyadic coping) and was scored directly. The Negative Dyadic Coping subscale contains 8 items measuring unsupportive, dismissive or hostile behaviours; all items were reverse‐scored using the formula reversed score = 6‐raw score, with higher reversed scores indicating fewer negative dyadic coping behaviours. The Cronbach's *α* of the questionnaire in Chinese was 0.84 (Xu et al. 2016).

### 2.3 Data Collection

The survey was conducted at the time of discharge for the hospitalized preterm infants. The parents of preterm infants who met the inclusion criteria were selected. To ensure that parents of preterm infants fully understood the study's purpose and voluntary participation, well‐trained research nurses briefed them on the meaning and purpose of all survey items and informed them that refusing to participate would not impact their treatment. After completing the survey, the questionnaires from both parents were matched using their baby's hospitalization identification.

### 2.4 Ethical Considerations

The Ethics Committee of Peking University Shenzhen Hospital granted ethical approval for the study (No. 2023 [019]). This study was prospectively registered at ClinicalTrials.gov (NCT05592210) with authorization from the original author for scale usage. Parental participation was preceded by informed consent. This study adhered to the Strengthening the Reporting of Observational Studies in Epidemiology guidelines (STROBE) (Ghaferi et al. 2021).

### 2.5 Data Analyses

Descriptive statistics, such as frequencies and percentages for categorical variables and means with standard deviations for continuous variables, were employed to summarize the demographic and sociological characteristics of the parents and infants. Normality of continuous variables was assessed using the Shapiro–Wilk test and visual inspection of histograms. When minor deviations from normality were detected, parametric analyses were retained due to their robustness under moderate violations given the sample size (Lumley et al. 2002). A paired *t*‐test and one‐way analysis of variance were employed to examine potential differences in parenting self‐efficacy, parenting stress and dyadic coping among parents. Pearson's correlation analysis was employed to investigate the correlations among the parental variables. Prior to interpreting the regression results, we assessed linearity, multicollinearity, homoscedasticity, normality and independence of errors using residual plots, VIF values (< 5), and the Durbin–Watson statistic (1.5–2.5). After all assumptions were satisfactorily met, hierarchical multiple linear regressions were performed for parents, with demographic variables entered in Block 1 and core predictors in Block 2, to assess correlates of parenting self‐efficacy, stress and dyadic coping.

The APIMeM (Ledermann et al. 2011) was employed to investigate the mediating roles of positive and negative coping models in the relationship between parenting stress and self‐efficacy. All variables were paired according to the couples included in the analysis. Parameters were estimated using the maximum likelihood (ML) method. Based on the above path analysis, a 95% confidence interval was estimated by conducting 2000 bootstrap repeated sampling. The model's fit was assessed using several indices with specific cut‐off values: *χ* ^2^/df less than 3, root mean square error of approximation (RMSEA) below 0.08, normed fit index (NFI), incremental fit index (IFI), and Tucker–Lewis index (TLI) all above 0.90 and comparative fit index (CFI) greater than 0.95 (Peugh et al. 2013).

EpiData 3.1 software was used for dual‐entry data input. This study had no missing data due to the online questionnaire collection and strict quality control of the offline questionnaire process. APIMeM was performed using Amos 26.0 (Arbuckle 2019). SPSS 26.0 (IBMCorp., Armonk, N.Y., USA) software was used for other data analyses (IBM Corp 2019). All tests were two‐tailed, and statistical significance was set at *p* < 0.05.

## 3 Results

A total of 165 questionnaires were distributed to parents of early preterm infants, for 330 questionnaires. A total of 285 (86.4%) questionnaires were returned, with 14 deemed invalid (4.9%). Finally, 240 questionnaires (120 pairs) were matched and analysed, accounting for 88.6% of the total questionnaires. Although the Shapiro–Wilk test indicated minor deviations from normality for several variables (*p* < 0.05), graphical inspection suggested approximate symmetry; thus, parametric analyses were retained. The demographic information of the 120 preterm infants included in this study is presented in Table 1. Their sex distribution was nearly equal (68 male infants vs. 52 female infants). A total of 70.0% (84/120) had a gestational age ranging from 32 to 37 weeks, and 61.7% (74/120) had a birth weight ranging from 1.5 to 2.5 kg. As shown in Table 2, the average age of mothers was 31.96 ± 4.05 years old, and the average age of fathers was 33.90 ± 4.26 years old. Mothers and fathers reported similar levels of parenting stress and self‐efficacy. However, significant differences were found in dyadic coping between mothers and fathers (131.59 vs. 124.48, *p* < 0.001), including positive coping (84.02 ± 11.18 vs. 79.43 ± 13.60, *p* = 0.002) and negative coping (32.37 ± 6.16 vs. 30.40 ± 7.31, *p* = 0.002).

**TABLE 1:** Demographic characteristics of the preterm infants (*N* = 120).

Variables		*N* (%)
Gestational age (week)	< 28	10 (8.3)
	28 ≤ GA < 32	26 (21.7)
	32 ≤ GA < 37	84 (70.0)
Gender	Male	68 (56.7)
	Female	52 (43.3)
Birth weight	> 2.5 kg	21 (17.5)
	1.5 kg < birth weight ≤ 2.5 kg	74 (61.7)
	1 kg < birth weight ≤ 1.5 kg	15 (12.5)
	≤ 1 kg	10 (8.4)
Mode of conception	Natural conception	78 (65.0)
	Artificial conception	1 (0.8)
	In vitro embryo transfer	41 (34.2)
Multiple births	Yes	38 (31.7)
	No	82 (68.3)

**TABLE 2:** Differences of the demographic and parenting variables between the preterm parents.

Variables	*N* (%)/*M* ± SD		*p*
	Mother (*N* = 120)	Father (*N* = 120)	
Age	31.96 ± 4.05	33.90 ± 4.26	< 0.001***
First child			
Yes	79 (65.80)	83 (69.20)	0.046*
No	41 (34.20)	37 (30.80)	
Occupation			
Health related occupations	6 (5)	5 (4.20)	0.001***
Full time mother/father	24 (20)	3 (2.50)	
Freelance	20 (16.70)	21 (17.50)	
Individual industrial and commercial households	4 (3.30)	4 (3.30)	
Others	66 (55)	87 (72.50)	
Educational level			
Junior high school and below	17 (14.20)	17 (14.20)	0.817
Senior high school	17 (14.20)	14 (11.70)	
Bachelor's degree/junior college	74 (61.70)	78 (65.0)	
Master degree or above	12 (10)	11 (9.20)	
Monthly income			
5 k or less	41 (34.20)	17 (14.20)	< 0.001***
5 k ~ 10 k	38 (31.70)	40 (33.30)	
10 k ~ 20 k	29 (24.20)	31 (25.80)	
20 k ~ 30 k	7 (5.80)	17 (14.20)	
30 k or above	5 (4.10)	15 (12.50)	
Parenting stress	**62.77 ± 19.13**	**64.18 ± 19.23**	**0.477**
Appearance and behaviour of infants	33.67 ± 10.16	34.76 ± 10.68	0.329
Change of parental role	15.67 ± 5.84	16.07 ± 5.56	0.483
Visual and auditory perception of parents in NICU	13.43 ± 5.10	13.35 ± 5.01	0.878
Dyadic coping	**131.59 ± 16.25**	**124.48 ± 18.58**	< 0.001***
*Positive coping*	**84.02 ± 11.18**	**79.43 ± 13.60**	0.002**
Self‐perceived stress communication	15.92 ± 2.13	14.15 ± 3.23	< 0.001***
Spouses perceive pressure communication	14.90 ± 2.66	14.38 ± 2.79	0.106
Self‐perceived support	18.93 ± 3.21	17.68 ± 3.79	0.002**
Spouse perceived support	19.19 ± 2.85	18.68 ± 3.01	0.129
Self‐perceived empowerment	7.55 ± 1.44	7.28 ± 1.59	0.151
Spouse perceived empowerment	7.53 ± 1.17	7.28 ± 1.30	0.064
Common coping	19.36 ± 2.82	18.41 ± 3.30	0.008**
*Negative coping*	**32.37 ± 6.16**	**30.40 ± 7.31**	0.002**
Self‐perceived negative coping	16.12 ± 3.30	14.81 ± 3.79	< 0.001***
Spouses perceive negative coping	16.25 ± 3.34	15.59 ± 3.86	0.054
Parenting self‐efficacy	**35.13 ± 7.59**	**34.51 ± 8.26**	**0.480**
Parenting	15.81 ± 4.37	15.98 ± 5.22	0.752
Support	12.89 ± 2.60	12.41 ± 2.65	0.076
Sense of competence	6.43 ± 1.54	6.12 ± 1.59	0.085

*Note:* Paired *t*‐test was used for continuous variables, and Wilcoxon signed‐rank test was used for categorical variables. *p < 0.05, **p < 0.01, ***p < 0.001.

The results of one‐way ANOVA in this study indicated that the parenting stress among mothers varied significantly based on educational level and family income (*p* < 0.05). Additionally, positive coping was significantly associated with personal monthly income and family income, whereas negative coping was significantly associated with firstborn status (*p* < 0.05). For fathers, parenting stress and positive dyadic coping were significantly associated with the infant's gestational age (*p* < 0.05), while negative dyadic coping was significantly associated with firstborn status, education level and monthly income (*p* < 0.05). No statistically significant differences were observed in other categories. Further details are provided in the Tables S1 and S2.

The results of the correlation analysis indicate that there are significant interrelationships among the three variables associated with parents of premature infants. Further details are provided in the Table S3.

Hierarchical regression analyses (Table 3) showed that, for mother's parenting stress, educational level (*β* = −0.24, *p* = 0.033, *R* ^2^ = 0.09) is a significant negative correlation factor, while the father's stress from observations of the infant's appearance and behaviour (*β* = 0.25, *p* = 0.004, *R* ^2^ = 0.15, Δ*R* ^2^ = 0.06) is a significant positive correlation factor. For father's parenting stress, mother's stress from change of parental role is a positive correlation factor, while the gestational weeks (*β* = −0.17, *p* = 0.047) are a negative correlation factor.

**TABLE 3:** Hierarchical regression results for all dependent variables.

Dependent variable/Model/Predictor	*B*	SE	*β*	*t*	*p*	*R* ^2^	△*R* ^2^
M‐parenting stress							
*Model 1 (Demographics)*						0.09	
Educational level	−5.57	2.58	−0.24	−2.16	0.033		
*Model 2 (Core predictors)*						0.15	0.06
F‐appearance and behaviour of infants	0.45	0.15	0.25	2.95	0.004**		
F‐parenting stress							
*Model 1 (Demographics)*						0.03	
Gestational age	−6.06	2.98	−0.18	−2.03	0.044		
*Model 2 (Core predictors)*						0.11	0.10
M‐change of parental role	1.13	0.32	0.31	3.56	0.001**		
Gestational age	−5.72	2.85	−0.17	−2.01	0.047		
M‐positive coping							
*Model 1 (Demographics)*						0.06	
M‐monthly income	3.09	1.33	0.26	2.32	0.020		
*Model 2 (Core predictors)*						0.18	0.12
M‐monthly income	3.07	1.25	0.26	2.45	0.016		
F‐negative coping	−0.49	0.17	−0.25	−2.92	0.004**		
M‐appearance and behaviour of infants	−0.32	0.11	−0.24	−2.77	0.006**		
F‐positive coping							
*Model 1 (Demographics)*						0.003	
(No significant predictors)							
BlockModel 2 (Core predictors)						0.06	0.07
M‐negative coping	−0.72	0.25	−0.26	−2.88	0.005**		
M‐negative coping							
*Model 1 (Demographics)*						0.01	
(No significant predictors)							
*Model 2 (Core predictors)*						0.17	0.16
F‐negative coping	0.35	0.07	0.42	4.84	0.000***		
F‐negative coping							
*Model 1 (Demographics)*						0.07	
First child	3.57	1.33	0.24	2.70	0.008**		
*Model 2 (Core predictors)*						0.25	0.19
First child	2.84	1.20	0.19	2.37	0.019		
M‐negative coping	0.45	0.10	0.38	4.69	0.000***		
F‐appearance and behaviour of infants	0.12	0.05	0.19	2.32	0.022		
M‐parenting self‐efficacy						0.27	—
M‐common coping	0.92	0.24	0.32	3.78	0.000***		
M‐appearance and behaviour of infants	−0.19	0.07	−0.24	−2.86	0.005**		
F‐visual and auditory perception of parents in NICU	0.36	0.12	0.25	3.09	0.003**		
F‐support	0.61	0.24	0.21	2.56	0.012		
F‐parenting self‐efficacy						0.32	—
F‐self perceived support	0.51	0.10	0.38	5.06	0.000***		
F‐appearance and behaviour of infants	−0.26	0.06	−0.33	−4.30	0.000***		
M‐parenting	0.42	0.14	0.23	3.02	0.003**		

*Note:* Model 1 includes the demographic variables related to the dependent variable in the single‐factor analysis. Only predictors with *p* < 0.05 in the final model are shown; non‐significant variables are omitted. Δ*R* ^2^ represents the change from Model 1 to Model 2; significance based on *F*‐change test.

Abbreviations: F, father; M, mother.

** *p* < 0.01.

*** *p* < 0.001.

Regarding coping strategies, mother's positive coping was associated with their own monthly income (*β* = 0.26, *p* = 0.016) and with stress from observations of the infant's appearance and behaviour (*β* = −0.25, *p* = 0.004). Father's negative coping was associated with first‐child status (*β* = 0.19, *p* = 0.019) and with their own stress from observations of the infant's appearance and behaviour (*β* = 0.19, *p* = 0.022). Notably, mother's and father's negative coping were mutually related to each other (*β* = 0.42 and 0.38, respectively, both *p* < 0.001). Moreover, positive coping in one parent was related to negative coping in the other parent (*β* = −0.26 and −0.25, *p* = 0.005 and 0.004, respectively), suggesting cross‐partner effects.

For parenting self‐efficacy, mother's common coping (*β* = 0.32, *p* < 0.001), father's NICU‐related perceptions (*β* = 0.25, *p* = 0.003), and father's support (*β* = 0.21, *p* = 0.012) were positively associated with their own self‐efficacy, whereas stress from observations of the infant's appearance and behaviour (*β* = −0.24, *p* = 0.005) was negatively associated (*R* ^2^ = 0.27). For fathers, perceived support from the partner (*β* = 0.38, *p* < 0.001) and mother's parenting (*β* = 0.23, *p* = 0.003) were positively related to their own self‐efficacy, while stress from observations of the infant's appearance and behaviour (*β* = −0.33, *p* < 0.001) was negatively related (*R* ^2^ = 0.32).

### 3.1 APIMeM Analyses

#### 3.1.1 Dyadic Positive Coping With Parenting Stress

**FIGURE 2:**
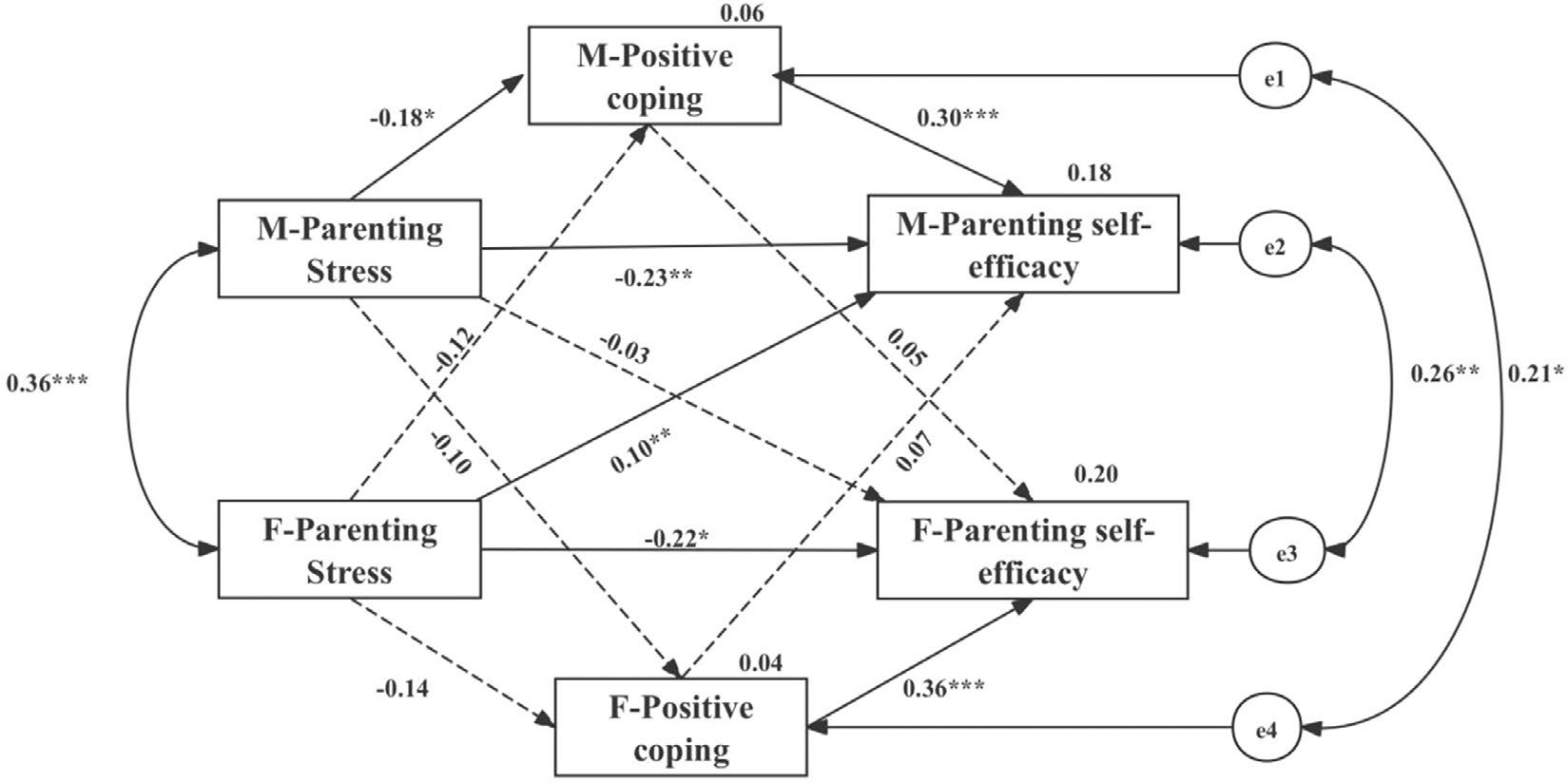
Path coefficients of parenting self‐efficacy with parenting stress mediated by positive coping. F, father; M, mother; solid lines indicate significant paths, and dashed lines indicate non‐significant paths; *p < 0.05, **p < 0.01, ***p < 0.001.

The APIMeM with parenting self‐efficacy as the outcome variable showed a good fit to the data (*χ* ^2^/df = 1.169, CFI = 0.992, TLI = 0.971, RMSEA = 0.038). As shown in Figure 2, the associations between parents' parenting stress and their parenting self‐efficacy were significant (mother: *β* = −0.23, *p* = 0.004; father: *β* = −0.22, *p* = 0.038). A mother's parenting stress was negatively related to her positive coping (*β* = −0.18, *p* = 0.026), but no significant effect of a father's parenting stress was found. Moreover, positive coping was positively correlated with each parent's parenting self‐efficacy (mother: *β* = 0.30, *p* < 0.001; father: *β* = 0.36, *p* < 0.001). Further examination of indirect effects revealed that mother's actor effects (*β* = −0.13, *p* = 0.002) and mother's partner effects (*β* = 0.08, *p* = 0.018) were present. Specifically, mother's parenting stress was indirectly and marginally related to their parenting self‐efficacy through their positive coping (*β* = −0.03, *p* = 0.01); the proportion of the total effect was 20.77%. The additional indices are presented in Table S4.

#### 3.1.2 Negative Coping With Parenting Stress

**FIGURE 3:**
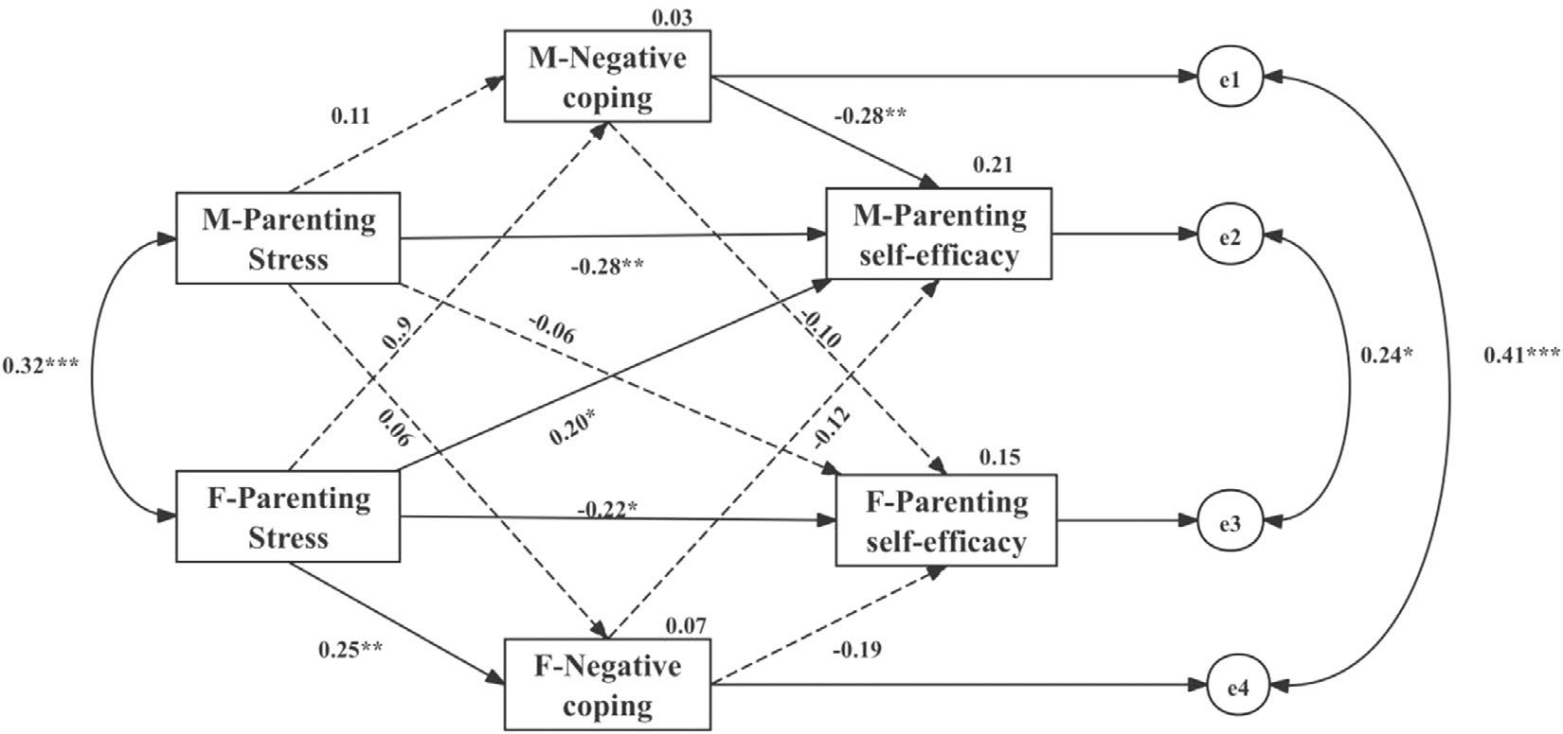
Path coefficients of parenting self‐efficacy with parenting stress mediated by Negative coping. F, father; M, mother; Solid lines indicate significant paths, and dashed lines indicate non‐significant paths; *p < 0.05, **p < 0.01, ***p < 0.001.

When negative coping served as the mediating variable, the model fits the data (*χ* ^2^/df = 1.268, CFI = 0.989, TLI = 0.959, RMSEA = 0.047). As shown in Figure 3, the results indicated that the relationships between parents' parenting stress and their parenting self‐efficacy were significant (mother: *β* = −0.28, *p* < 0.001; father: *β* = −0.22, *p* = 0.016). A father's parenting stress was positively correlated with his negative coping (*β* = 0.25, *p* = 0.007). A mother's negative coping was negatively correlated with parenting self‐efficacy (*β* = −0.28, *p* = 0.002). For the mother's partner effect, the association between the father's parenting stress and the mother's parenting self‐efficacy was significant (*β* = 0.20, *p* = 0.021). However, the mediating effect of negative coping was not significant for either actor or partner effects. Additional indices are presented in Table S5 with CIs.

## 4 Discussion

This study provides novel insights into the actor and partner effects of parenting stress on the parenting self‐efficacy of preterm infants' parents and the mediating effect of dyadic coping using the APIMeM approach. These findings underscore that parenting stress was negatively associated with self‐efficacy in both mothers and fathers; positive dyadic coping partially mediated the association between stress and self‐efficacy for mothers but not for fathers. Meanwhile, clear gender differences were observed in dyadic coping patterns. The results indicate a direct and indirect relationship between mothers' parenting stress and self‐efficacy. Together, these findings highlight the interdependent nature of parental adjustment in the NICU context and underscore the importance of examining stress and coping at the couple level.

Our results are consistent with dyadic stress theories rooted in general systems theory and the transactional model of stress and coping (Landolt et al. 2023). Bodenmann's (2008) systemic‐transactional model conceptualizes coping as an interpersonal process in which partners jointly appraise and respond to shared stressors. Preterm birth and NICU hospitalization represent profound shared stress experiences, requiring coordinated adaptation rather than isolated individual responses. Similarly, Lyons' Theory of Dyadic Illness Management (Lyons and Lee 2018) emphasizes that illness management and psychological adjustment are inherently dyadic, characterized by interdependent outcomes and shared meaning‐making processes. The actor and partner effects identified in this study support these frameworks, demonstrating that parental stress and coping processes operate within a relational system rather than independently.

Although the proportion of variance explained in parenting stress was modest, similar findings have been reported in NICU populations (Pavlyshyn et al. 2024), reflecting the multifactorial nature of stress responses in this setting. Demographic analyses revealed that lower educational level and family income were associated with higher maternal stress, whereas lower gestational age was associated with greater paternal stress. These patterns are consistent with prior evidence suggesting that socioeconomic vulnerability and infant medical fragility heighten psychological burden (Bua et al. 2024). Mothers may experience greater anxiety related to caregiving competence and financial strain (Lakshmanan et al. 2022), whereas fathers may focus more on infant prognosis and medical stability. Such differences in stress appraisal may shape distinct coping pathways within couples.

In the analysis of parenting self‐efficacy, studies show that parents of preterm infants demonstrate low parenting self‐efficacy, which is consistent with other studies (Grunberg et al. 2025). Parenting stress was negatively associated with self‐efficacy in both parents, echoing findings from systematic reviews (Fang et al. 2021). However, the mechanisms linking stress and self‐efficacy differed by coping type and gender. In the positive coping model, mothers' positive coping significantly mediated the association between their stress and self‐efficacy, whereas no mediation effect was observed for fathers. Positive coping behaviours, such as mutual support and collaborative problem‐solving, may enhance mothers' sense of competence and buffer stress‐related self‐doubt (Rusu et al. 2018). In contrast, although fathers' stress was associated with higher negative coping, negative coping did not mediate the stress–self‐efficacy relationship for either parent. Instead, mothers' negative coping was directly associated with lower self‐efficacy. These findings suggest that positive and negative coping function through distinct mechanisms that constructive dyadic engagement may actively strengthen maternal confidence, whereas negative coping reflects relational strain without necessarily transmitting stress effects.

Interestingly, an unexpected positive partner effect was observed, whereby higher paternal stress was associated with higher maternal self‐efficacy. This finding should not be interpreted as indicating beneficial effects of paternal stress. Rather, it may reflect increased paternal engagement and shared responsibility in caregiving, even when accompanied by psychological burden. Prior intervention research has shown that father's active involvement can enhance maternal adjustment (Hadian Shirazi et al. 2022). Thus, paternal stress may sometimes signal involvement rather than disengagement, highlighting the complexity of dyadic interdependence in the NICU context.

In this study, parents reported moderate levels of dyadic coping, consistent with previous findings during the transition to parenthood (Molgora et al. 2019). However, significant gender differences were identified. Mothers reported higher overall dyadic coping, including stress communication and supportive behaviours. Traditional gender norms in China continue to position mothers as primary caregivers and fathers as financial providers (Liu et al. 2024). These culturally embedded expectations may influence emotional expression, help‐seeking behaviours, and involvement in neonatal care. Mothers often assume greater responsibility for direct infant care in the NICU, which may heighten both stress exposure and engagement in coping processes (Loewenstein et al. 2021). Fathers, in contrast, may prioritize economic roles or suppress emotional expression, limiting visible supportive behaviours (Alnuaimi and Tluczek 2022).

Structural factors may further reinforce these gendered patterns. NICU visiting policies and infection control measures frequently restrict parental presence and bedside participation (Yue et al. 2020). Such constraints may disproportionately limit fathers' opportunities for hands‐on caregiving, including kangaroo care, which remains more common among mothers (Dong et al. 2025). Reduced participation may impede fathers' development of caregiving confidence and active dyadic coping. At the same time, China is undergoing gradual shifts toward more gender‐equitable parenting (Guo 2025), suggesting that traditional and emerging role expectations coexist. These cultural and institutional influences likely shape the observed gender differences in coping and stress responses.

Beyond gender norms and structural constraints, the quality of staff–parent relationships also plays a critical role in shaping parental adjustment. Effective communication and collaborative decision‐making can foster parents' sense of partnership and legitimacy in care (Thébaud et al. 2022). Tools designed to strengthen staff–parent relationships, such as structured guidance programmes in neonatal units, have been shown to enhance parental engagement and care quality (Eeles and Gibbs 2020). A supportive, family‐centred NICU environment may therefore facilitate more balanced dyadic coping and mitigate gender disparities in involvement.

Taken together, these findings emphasize that parenting stress and self‐efficacy among parents of preterm infants are embedded within relational, cultural and structural contexts. Positive dyadic coping appears particularly protective for mothers, whereas paternal involvement—even when accompanied by stress—may exert indirect benefits within the couple system. Interventions that focus exclusively on individual parents may overlook these interdependent processes.

### 4.1 Implications for Practice

Based on these findings, NICU teams should adopt gender‐sensitive and family‐centred approaches that assess both parents' stress and coping patterns. Because positive coping demonstrated protective effects for mothers, interventions that promote collaborative problem‐solving, guided communication and shared caregiving experiences may strengthen maternal self‐efficacy. For fathers, strategies that clarify caregiving roles, facilitate structured involvement in infant care and reduce avoidant coping may enhance engagement and confidence. NICU nurses may also provide tailored education for parents with limited health literacy and facilitate access to financial or social support resources.

Importantly, as negative coping did not mediate the stress–self‐efficacy association, clinical efforts may be more effectively directed toward reducing parenting stress and promoting constructive dyadic coping rather than focusing solely on minimizing negative behaviours. Culturally tailored interventions that acknowledge traditional parental roles while supporting evolving co‐parenting dynamics are warranted. By addressing both individual and relational processes, NICU care teams can foster more balanced parental adaptation and strengthen family resilience in the context of preterm birth.

### 4.2 Limitations and Directions for Future Research

This study has several limitations that should be considered when interpreting the findings. First, the sample size (*N* = 120 dyads), while acceptable for detecting direct and simple indirect effects in the APIMeM based on post hoc Monte Carlo simulations, is below the generally recommended threshold of ≥ 200 dyads for obtaining stable and reliable estimates in complex dyadic mediation models (Ledermann et al. 2022). Therefore, mediation estimates total and indirect effects should be interpreted with appropriate caution and considered preliminary pending replication in larger samples. Second, the cross‐sectional design cannot capture dynamic changes in parenting stress, self‐efficacy or coping over time. Third, the use of convenience sampling and self‐reported measures may introduce selection and response biases. Parents who agreed to participate may have been more motivated or less stressed than those who declined, and self‐reported stress and coping may be influenced by social desirability or recall bias. Fourth, the modest *R* ^2^ for parenting stress suggests that other unmeasured factors not included in our model may also contribute to parental stress. Fifth, the sample was predominantly drawn from economically affluent regions, consisted mainly of first‐time parents and included a higher proportion of moderately preterm infants, which may limit the generalizability of our findings. Future longitudinal studies with larger, more diverse samples across preterm subgroups and parental backgrounds are needed to replicate and extend these results.

## 5 Conclusion

This study highlighted the connections between parenting stress, parenting self‐efficacy, and dyadic coping in Chinese parents of preterm infants. The findings highlight the important role that positive coping mechanisms have in reducing parenting stress and raising parenting self‐efficacy. Moreover, the results emphasize the interconnectedness of parental dynamics and the need for interventions that take into account both dyadic and individual aspects. In addition, findings suggest that parenting self‐efficacy may be impacted by differences in parents' communication and support methods during stressful times. As a result, nurses and clinicians should prioritize fostering effective stress communication, collaborative positive coping methods and co‐parenting self‐efficacy in parental education for preterm infants. Future studies should build on these findings by focusing on longitudinal impacts, using objective measures and examining additional background characteristics to help develop targeted, evidence‐based interventions.

## Author Contributions

Yuying Mo had primary responsibility for protocol development, patient screening, enrollment, outcome assessment, preliminary data analysis and writing the manuscript. Wanxiang He participated in the development of the protocol and analytical framework for the study and contributed to the writing of the manuscript. Xiaojuan Liu contributed to the writing of the manuscript and was responsible for patient screening. Wei Xia supervised the design and execution of the study, performed the final data analyses and contributed to the writing of the manuscript. All authors read and approved the final manuscript.

## Funding

This work was supported by the Peking University Shenzhen Hospital [Grant numbers LCYJ2022038].

## Ethics Statement

The Ethics Committee of Peking University Shenzhen Hospital granted ethical approval for the study (No. 2023 [019]).

## Consent

Informed consent was obtained from all individual participants included in the study.

## Conflicts of Interest

The authors declare no conflicts of interest.

## Supporting information


**Appendix S1:** Table S1. Differences in parenting self‐efficacy, parenting stress, positive coping, and negative coping among mothers of preterm infants with varied demographic characteristics.
**Table S2:** Differences in parenting self‐efficacy, parenting stress, positive coping, and negative coping among fathers of preterm infants with varied demographic characteristics.
**Table S3:** Bivariate coefficients among study variables.
**Table S4:** Positive coping effects for actor‐partner interdependence mediation models.
**Table S5:** Negative coping effects for actor‐partner interdependence mediation models.

## Data Availability

The data that support the findings of this study are available from the corresponding author upon reasonable request.
