# Supplementary material for: Parenting Stress, Parenting Self‐Efficacy and Dyadic Coping in Parents of Preterm Infants in China: A Dyadic Analysis
Source: Nurs Open. 2026 Jul 17;13(7):e70700. doi: 10.1002/nop2.70700 (PMC13377212; doi:10.1002/nop2.70700)
Supplement: Supplementary file 1 — Appendix S1: Table S1. Differences in parenting self‐efficacy, parenting stress, positive coping, and negative coping among mothers of preterm infants with varied demographic characteristics. Table S2: Differences in parenting self‐efficacy, parenting stress, positive coping, and negative coping among fathers of preterm infants with varied demographic characteristics. Table S3: Bivariate coefficients among study variables. Table S4: Positive coping effects for actor‐partner interdependence mediation models. Table S5: Negative coping effects for actor‐partner interdependence mediation models. [file NOP2-13-e70700-s001.docx]

**Table S1 Differences in Parenting Self-Efficacy, Parenting Stress, Positive Coping, and Negative Coping Among Mothers of Preterm Infants with Varied Demographic Characteristics**

| **Mothers Variable** | **Parenting Stress**  **（M±SD）** | ***P*** | **Positive coping （M±SD）** | ***P*** | **Negative coping（M±SD）** | ***P*** | **Parenting self-efficacy（M±SD）** | ***P*** |
| --- | --- | --- | --- | --- | --- | --- | --- | --- |
| **Length of hospital stay for infants** |  | 0.192 |  | 0.080 |  | 0.559 |  | 0.951 |
| ≤ 7days | 61.71 ± 19.44 |  | 81.97 ± 9.84 |  | 31.43 ± 6.39 |  | 35.00 ± 8.06 |  |
| 7 << LOS ≤ 30 days | 60.25 ± 18.56 |  | 86.63 ± 12.02 |  | 32.85 ± 6.31 |  | 35.37 ± 7.88 |  |
| > 30 days | 67.85 ± 19.28 |  | 82.06 ± 10.57 |  | 32.61 ± 5.74 |  | 34.88 ± 6.78 |  |
| **Age** |  | 0.852 |  | 0.672 |  | 0.253 |  | 0.933 |
| 18 ≤ Age < 30 | 62.88 ± 17.75 |  | 84.05 ± 9.19 |  | 31.41 ± 6.62 |  | 34.93 ± 9.19 |  |
| 30 ≤ Age < 40 | 62.51 ± 20.05 |  | 84.18 ± 12.23 |  | 32.99 ± 5.86 |  | 35.27 ± 6.65 |  |
| ≥ 40 | 70.50 ± 16.26 |  | 77.00 ± 5.66 |  | 28.00 ± 7.07 |  | 33.50 ± 10.61 |  |
| Gender of preterm infants |  | 0.792 |  | 0.708 |  | 0.234 |  | 0.570 |
| male | 63.18 ± 18.20 |  | 84.35 ± 10.72 |  | 31.78 ± 6.49 |  | 35.47 ± 7.05 |  |
| female | 62.23 ± 20.44 |  | 83.58 ± 11.85 |  | 33.13 ± 5.68 |  | 34.67 ± 8.30 |  |
| **Multiple births** |  | 0.802 |  | 0.537 |  | 0.926 |  | 0.743 |
| Yes | 63.42 ± 21.39 |  | 84.95 ± 10.53 |  | 32.29 ± 6.16 |  | 34.79 ± 7.52 |  |
| No | 62.46 ± 18.11 |  | 83.59 ± 11.51 |  | 32.40 ± 6.20 |  | 35.28 ± 7.66 |  |
| **First child or not** |  | 0.770 |  | 0.188 |  | 0.049 |  | 0.062 |
| Yes | 63.14 ± 18.95 |  | 84.99 ± 11.29 |  | 33.16 ± 5.53 |  | 34.18 ± 7.37 |  |
| No | 62.05 ± 19.68 |  | 82.15 ± 10.85 |  | 30.83 ± 7.05 |  | 36.95 ± 7.77 |  |
| **Gestational age (week)** |  | 0.170 |  | 0.416 |  | 0.791 |  | 0.540 |
| <28 | 67.20 ± 20.30 |  | 79.60 ± 14.67 |  | 33.20 ± 6.75 |  | 36.80 ± 3.91 |  |
| 28≤GA<32 | 68.04 ± 20.02 |  | 83.92 ± 9.27 |  | 32.85 ± 5.45 |  | 33.88 ± 7.37 |  |
| 32≤GA<37 | 60.61 ± 18.53 |  | 84.57 ± 11.28 |  | 32.12 ± 6.35 |  | 35.31 ± 7.98 |  |
| **Birth weight** |  | 0.630 |  | 0.133 |  | 0.235 |  | 0.820 |
| >2.5kg | 58.62 ± 22.71 |  | 85.78 ± 11.05 |  | 33.22 ± 6.45 |  | 36.52 ± 6.35 |  |
| 1.5kg<birth weight≤2.5kg | 62.51 ± 17.36 |  | 82.93 ± 11.3 |  | 31.33 ± 4.85 |  | 35.14 ± 8.21 |  |
| 1kg<birth weight≤1.5kg | 66.13 ± 22.19 |  | 74.60 ± 15.26 |  | 33.20 ± 4.66 |  | 33.80 ± 7.48 |  |
| ≤1kg | 65.00 ± 19.89 |  | 82.80 ± 10.43 |  | 32.80 ± 8.2 |  | 35.20 ± 3.27 |  |
| Natural Conception |  | 0.283 |  | 0.667 |  | 0.936 |  | 0.363 |
| Yes | 61.38 ± 17.07 |  | 83.69 ± 11.46 |  | 32.33 ± 5.88 |  | 35.59 ± 7.43 |  |
| No | 65.33 ± 22.45 |  | 84.62 ± 10.74 |  | 32.43 ± 6.73 |  | 34.26 ± 7.89 |  |
| **Occupation** |  | 0.160 |  | 0.475 |  | 0.171 |  | 0.080 |
| Health related occupations | 64.33 ± 14.61 |  | 83.00 ± 13.21 |  | 29.67 ± 5.99 |  | 28.33 ± 9.56 |  |
| full time mother/father | 61.25 ± 19.15 |  | 83.71 ± 9.53 |  | 30.25 ± 7.65 |  | 34.46 ± 10.50 |  |
| freelance | 72.55 ± 23.49 |  | 83.05 ± 9.38 |  | 31.85 ± 6.82 |  | 34.35 ± 7.28 |  |
| Individual industrial and commercial households | 57.25 ± 20.85 |  | 74.75 ± 14.43 |  | 33.00 ± 4.55 |  | 41.00 ± 4.24 |  |
| others | 60.55 ± 17.46 |  | 85.08 ± 11.88 |  | 33.50 ± 5.27 |  | 35.86 ± 5.99 |  |
| **Educational level** |  | 0.010 |  | 0.296 |  | 0.298 |  | 0.970 |
| Junior high school and below | 71.88 ± 19.53 |  | 79.94 ± 9.8 |  | 30.76 ± 6.89 |  | 35.12 ± 9.20 |  |
| Senior high school | 72.47 ± 22.59 |  | 82.65 ± 13.1 |  | 30.71 ± 8.81 |  | 34.82 ± 9.82 |  |
| Bachelor's degree ‎/ junior college | 59.18 ± 17.95 |  | 84.78 ± 10.36 |  | 33.18 ± 5.18 |  | 35.34 ± 6.86 |  |
| Master degree or above | 58.25 ± 11.81 |  | 87.00 ± 14.41 |  | 32.00 ± 6.18 |  | 34.25 ± 6.85 |  |
| **Monthly income** |  | 0.176 |  | <0.001 |  | 0.599 |  | 0.526 |
| 5k or less | 66.90 ± 21.52 |  | 80.71 ± 8.75 |  | 32.02 ± 5.92 |  | 34.29 ± 9.10 |  |
| 5k～30k | 61.04 ± 17.44 |  | 84.8 ± 11.46 |  | 32.38 ± 6.40 |  | 35.39 ± 6.84 |  |
| 30k or above | 54.40 ± 19.60 |  | 99.6 ± 11.33 |  | 35.00 ± 4.80 |  | 38.00 ± 3.54 |  |
| **Family income** |  | 0.010 |  | 0.009 |  | 0.264 |  | 0.240 |
| 50k or less | 76.29 ± 18.35 |  | 82.93 ± 5.70 |  | 30.29 ± 7.08 |  | 32.93 ± 13.25 |  |
| 50k～200k | 63.35 ± 20.68 |  | 80.94 ± 10.8 |  | 32.09 ± 6.87 |  | 34.52 ± 6.34 |  |
| 200k or above | 58.52 ± 15.96 |  | 87.5 ± 11.81 |  | 33.21 ± 4.98 |  | 36.35 ± 6.68 |  |

**Table S2 Differences in Parenting Self-Efficacy, Parenting Stress, Positive Coping, and Negative Coping Among Fathers of Preterm Infants with Varied Demographic Characteristics**

| **Fathers Variable** | **Parenting Stress**  **（M±SD）** | ***P*** | **Positive coping （M±SD）** | ***P*** | **Negative coping（M±SD）** | ***P*** | **Parenting self-efficacy（M±SD）** | ***P*** |
| --- | --- | --- | --- | --- | --- | --- | --- | --- |
| **Length of hospital stay for infants** |  | 0.110 |  | 0.221 |  | 0.897 |  | 0.750 |
| ≤ 7days | 61.57 ± 17.94 |  | 77.46 ± 12.20 |  | 29.94 ± 6.75 |  | 34.06 ± 9.82 |  |
| 7 < LOS ≤ 30 days | 62.10 ± 19.60 |  | 81.90 ± 12.23 |  | 30.69 ± 7.19 |  | 35.17 ± 7.71 |  |
| > 30 days | 70.21 ± 19.22 |  | 77.64 ± 16.55 |  | 30.42 ± 8.20 |  | 33.94 ± 7.45 |  |
| **Age** |  | 0.450 |  | 0.681 |  | 0.282 |  | 0.680 |
| 18 ≤ Age < 30 | 64.48 ± 20.21 |  | 81.44 ± 12.57 |  | 28.78 ± 7.07 |  | 35.56 ± 9.80 |  |
| 30 ≤ Age < 40 | 63.31 ± 18.58 |  | 78.91 ± 14.30 |  | 31.08 ± 7.13 |  | 34.08 ± 7.93 |  |
| ≥ 40 | 72.38 ± 23.26 |  | 78.25 ± 9.16 |  | 28.63 ± 9.64 |  | 35.50 ± 6.46 |  |
| Gender of preterm infants |  | 0.393 |  | 0.368 |  | 0.741 |  | 0.320 |
| male | 62.83 ± 19.62 |  | 80.43 ± 14.03 |  | 30.60 ± 7.76 |  | 35.18 ± 9.01 |  |
| female | 65.87 ± 18.78 |  | 78.17 ± 13.06 |  | 30.15 ± 6.76 |  | 33.66 ± 7.20 |  |
| **Multiple births** |  | 0.810 |  | 0.486 |  | 0.426 |  | 0.808 |
| Yes | 63.55 ± 21.74 |  | 80.71 ± 14.27 |  | 31.18 ± 7.80 |  | 34.24 ± 8.57 |  |
| No | 64.46 ± 18.09 |  | 78.84 ± 13.33 |  | 30.04 ± 7.09 |  | 34.63 ± 8.17 |  |
| **First child or not** |  | 0.241 |  | 0.461 |  | 0.005 |  | 0.518 |
| Yes | 62.80 ± 17.67 |  | 80.05 ± 14.59 |  | 31.65 ± 6.78 |  | 34.18 ± 8.42 |  |
| No | 67.27 ± 22.30 |  | 78.05 ± 11.14 |  | 27.59 ± 7.74 |  | 35.24 ± 7.96 |  |
| **Gestational age (week)** |  | 0.039 |  | 0.028 |  | 0.702 |  | 0.655 |
| <28 | 78.60 ± 17.73 |  | 68.7 ± 18.52 |  | 28.70 ± 7.53 |  | 32.40 ± 6.55 |  |
| 28≤GA<32 | 64.58 ± 18.08 |  | 81.58 ± 12.92 |  | 31.00 ± 8.06 |  | 34.15 ± 7.73 |  |
| 32 ≤ GA<37 | 62.33 ± 19.21 |  | 80.05 ± 12.73 |  | 30.42 ± 7.10 |  | 34.87 ± 8.63 |  |
| **Birth weight** |  | 0.114 |  | 0.389 |  | 0.814 |  | 0.970 |
| >2.5kg | 57.81 ± 20.82 |  | 76.86 ± 12.74 |  | 29.05 ± 6.26 |  | 34.62 ± 6.32 |  |
| 1.5kg<birth weight≤2.5kg | 64.85 ± 18.28 |  | 81.27 ± 11.69 |  | 30.68 ± 7.43 |  | 34.80 ± 8.94 |  |
| 1kg<birth weight≤1.5kg | 61.13 ± 18.69 |  | 74.53 ± 21.17 |  | 31.73 ± 7.97 |  | 34.00 ± 8.87 |  |
| ≤1kg | 79.20 ± 15.09 |  | 79.80 ± 18.10 |  | 29.40 ± 7.33 |  | 32.60 ± 3.85 |  |
| **Occupation** |  | 0.271 |  | 0.052 |  | 0.115 |  | 0.653 |
| Health related occupations | 62.40 ± 21.04 |  | 88.00 ± 1.87 |  | 37.80 ± 2.28 |  | 39.00 ± 3.08 |  |
| full time mother/father | 48.67 ± 16.86 |  | 66.67 ± 3.06 |  | 25.33 ± 2.31 |  | 38.00 ± 4.00 |  |
| freelance | 71.29 ± 21.28 |  | 74.14 ± 11.99 |  | 29.95 ± 7.13 |  | 34.95 ± 7.82 |  |
| Individual industrial and commercial households | 65.25 ± 27.99 |  | 74.75 ± 11.18 |  | 33.25 ± 6.90 |  | 35.50 ± 6.45 |  |
| others | 63.05 ± 18.16 |  | 80.87 ± 14.07 |  | 30.13 ± 7.45 |  | 33.98 ± 8.72 |  |
| **Educational level** |  | 0.425 |  | 0.498 |  | 0.049 |  | 0.807 |
| Junior high school and below | 64.47 ± 19.24 |  | 79.24 ± 11.87 |  | 26.24 ± 9.31 |  | 33.41 ± 6.31 |  |
| Senior high school | 71.14 ± 24.69 |  | 77.79 ± 12.27 |  | 29.14 ± 8.24 |  | 35.93 ± 8.42 |  |
| Bachelor's degree ‎/ junior college | 63.64 ± 17.99 |  | 78.94 ± 14.47 |  | 31.51 ± 6.29 |  | 34.32 ± 8.72 |  |
| Master degree or above | 58.64 ± 20.52 |  | 85.36 ± 11.19 |  | 30.55 ± 7.92 |  | 35.73 ± 8.05 |  |
| **Monthly income** |  | 0.089 |  | 0.434 |  | 0.006 |  | 0.545 |
| 5k or less | 73.53 ± 21.05 |  | 77.06 ± 10.92 |  | 25.35 ± 9.43 |  | 32.82 ± 8.43 |  |
| 5k～30k | 62.91 ± 19.11 |  | 79.25 ± 14.16 |  | 31.43 ± 6.46 |  | 35.00 ± 8.58 |  |
| 30k or above | 61.00 ± 15.54 |  | 83.20 ± 13.00 |  | 30.07 ± 7.40 |  | 33.53 ± 5.99 |  |
| **Family income** |  | 0.628 |  | 0.464 |  | 0.054 |  | 0.695 |
| 50k or less | 68.57 ± 18.48 |  | 75.93 ± 12.47 |  | 26.86 ± 9.52 |  | 33.00 ± 7.41 |  |
| 50k～200k | 64.23 ± 21.40 |  | 80.83 ± 11.87 |  | 29.85 ± 7.14 |  | 35.08 ± 8.73 |  |
| 200k or above | 62.96 ± 17.22 |  | 78.96 ± 15.43 |  | 31.89 ± 6.52 |  | 34.34 ± 8.09 |  |

**Table S3 Bivariate Coefficients among Study Variables.**

| Variable | | 1 | 2 | 3 | 4 | 5 | 6 | 7 | 8 | 9 | 10 |
| --- | --- | --- | --- | --- | --- | --- | --- | --- | --- | --- | --- |
| Mothers | 1. Parenting Stress | 1 |  |  |  |  |  |  |  |  |  |
|  | 1. Parenting self-efficacy | -0.26** | 1 |  |  |  |  |  |  |  |  |
|  | 1. Dyadic coping | -0.28** | 0.41** | 1 |  |  |  |  |  |  |  |
|  | 1. Positive coping | -0.27** | 0.33** | 0.94** | 1 |  |  |  |  |  |  |
|  | 1. Negative coping | -0.10 | 0.35** | 0.64** | 0.36** | 1 |  |  |  |  |  |
| Fathers | 1. Parenting Stress | 0.36** | 0.07 | -0.21* | -0.24** | -0.03 | 1 |  |  |  |  |
|  | 1. Parenting self-efficacy | -0.19* | 0.28** | 0.29** | 0.25** | 0.25** | -0.27** | 1 |  |  |  |
|  | 1. Dyadic coping | -0.09 | 0.18 | 0.37** | 0.31** | 0.32** | -0.13 | 0.48** | 1 |  |  |
|  | 1. Positive coping | -0.07 | 0.08 | 0.24** | 0.15 | 0.23* | -0.10 | 0.41** | 0.92** | 1 |  |
|  | 1. Negative coping | -0.08 | 0.27** | 0.54** | 0.28** | 0.49** | -0.16 | 0.32** | 0.54** | 0.19* | 1 |

* *p* < .05.

** *p* < .0

**Pearson linear correlation results**

Table 4 presents the bivariate correlations among study variables. The parenting stress of mothers was negatively correlated with their parenting self-efficacy (r=-0.26, P=0.004) and dyadic coping (r=-0.28, P=0.002), and the parenting self-efficacy was positively correlated with dyadic coping (r=0.41, P<0.001). The parenting stress of fathers was negatively correlated with their parenting self-efficacy (r=-0.27, P=0.003), and the parenting self-efficacy was positively correlated with dyadic coping (r=0.48, P<0.001). Parenting stress (r=0.36, P < 0.001), parenting self-efficacy (r=0.28, P=0.002) and dyadic coping (r=0.37, P < 0.001) between parents were all positively correlated. Mothers' parenting stress was negatively correlated with fathers' parenting self-efficacy (r=-0.19, P =0.036), and mothers' dyadic coping was positively correlated with fathers' parenting self-efficacy (r=0.29, P = 0.001).

**Table S4 Positive coping Effects for Actor-Partner Interdependence Mediation Models.**

| Effect | effect size | 95%CIs | | *P* |
| --- | --- | --- | --- | --- |
|  |  | upper | lower |  |
| M-Stress→M-self-efficacy |  |  |  |  |
| Total effect | -0.13 | -0.22 | -0.05 | 0.002 |
| Total indirect effect | -0.03 | -0.06 | -0.01 | 0.011 |
| M-Stress→M-Positive coping→M-self-efficacy | -0.02 | -0.06 | -0.01 | 0.010 |
| M-Stress→F-Positive coping→M-self-efficacy | 0 | -0.01 | 0.01 | 0.874 |
| Direct effect | -0.10 | -0.19 | -0.03 | 0.005 |
| F-Stress→F-self-efficacy |  |  |  |  |
| Total effect | -0.10 | -0.18 | -0.02 | 0.010 |
| Total indirect effect | -0.02 | -0.07 | 0.01 | 0.262 |
| F-Stress→F-Positive coping→F-self-efficacy | -0.01 | -0.07 | 0.01 | 0.311 |
| F-Stress→M-Positive coping→F-self-efficacy | -0.01 | -0.03 | 0.01 | 0.250 |
| Direct effect | -0.08 | -0.16 | -0.004 | 0.038 |
| M-Stress→F-self-efficacy |  |  |  |  |
| Total effect | -0.05 | -0.14 | 0.02 | 0.119 |
| Total indirect effect | -0.02 | -0.06 | 0 | 0.059 |
| M-Stress→M-Positive coping→F-self-efficacy | 0 | -0.02 | 0.01 | 0.822 |
| M-Stress→F-Positive coping→F-self-efficacy | -0.02 | -0.05 | 0 | 0.054 |
| Direct effect | -0.03 | -0.12 | 0.04 | 0.313 |
| F-Stress→M-self-efficacy |  |  |  |  |
| Total effect | 0.08 | 0.02 | 0.15 | 0.018 |
| Total indirect effect | -0.01 | -0.05 | 0.02 | 0.394 |
| F-Stress→F-Positive coping→M-self-efficacy | -0.01 | -0.03 | 0.01 | 0.248 |
| F-Stress→M-Positive coping→M-self-efficacy | -0.01 | -0.05 | 0.02 | 0.648 |
| Direct effect | 0.10 | 0.04 | 0.16 | 0.007 |

NOTES. F: Father; M: Mother

**Table S5 Negative coping Effects for Actor-Partner Interdependence Mediation Models.**

| Effect | effect size | 95%CIs | | *P* |
| --- | --- | --- | --- | --- |
|  |  | upper | lower |  |
| M-Stress→M-self-efficacy |  |  |  |  |
| Total effect | -0.134 | -0.215 | -0.054 | 0.001 |
| Total indirect effect | -0.017 | -0.054 | 0.007 | 0.196 |
| M-Stress→M-Negative coping→M-self-efficacy | -0.013 | -0.05 | 0.009 | 0.286 |
| M-Stress→F-Negative coping→M-self-efficacy | -0.004 | -0.019 | 0.006 | 0.515 |
| Direct effect | -0.117 | -0.192 | -0.041 | 0.003 |
| F-Stress→F-self-efficacy |  |  |  |  |
| Total effect | -0.122 | -0.204 | -0.047 | 0.001 |
| Total indirect effect | -0.023 | -0.055 | 0.006 | 0.115 |
| F-Stress→F-Negative coping→F-self-efficacy | -0.021 | -0.05 | 0.003 | 0.091 |
| F-Stress→M-Negative coping→F-self-efficacy | -0.003 | -0.018 | 0.011 | 0.728 |
| Direct effect | -0.099 | -0.19 | -0.02 | 0.015 |
| M-Stress→F-self-efficacy |  |  |  |  |
| Total effect | -0.048 | -0.141 | 0.038 | 0.280 |
| Total indirect effect | -0.021 | -0.063 | 0.011 | 0.195 |
| M-Stress→M-Negative coping→F-self-efficacy | -0.013 | -0.044 | 0.01 | 0.301 |
| M-Stress→F-Negative coping→F-self-efficacy | -0.008 | -0.048 | 0.018 | 0.602 |
| Direct effect | -0.028 | -0.114 | 0.053 | 0.515 |
| F-Stress→M-self-efficacy |  |  |  |  |
| Total effect | 0.075 | 0.008 | 0.137 | 0.027 |
| Total indirect effect | -0.011 | -0.034 | 0.006 | 0.200 |
| F-Stress→F-Negative coping→M-self-efficacy | -0.005 | -0.024 | 0.005 | 0.420 |
| F-Stress→M-Negative coping→M-self-efficacy | -0.006 | -0.024 | 0.007 | 0.381 |
| Direct effect | 0.086 | 0.023 | 0.147 | 0.008 |
